# Antimicrobial Photodynamic Inactivation Affects the Antibiotic Susceptibility of *Enterococcus* spp. Clinical Isolates in Biofilm and Planktonic Cultures

**DOI:** 10.3390/biom11050693

**Published:** 2021-05-05

**Authors:** Agata Woźniak, Beata Kruszewska, Michał Karol Pierański, Michał Rychłowski, Mariusz Grinholc

**Affiliations:** 1 Laboratory of Molecular Diagnostics, Intercollegiate Faculty of Biotechnology, University of Gdansk and Medical University of Gdansk, 80-307 Gdansk, Poland; agata.wozniak@phdstud.ug.edu.pl (A.W.); beata.kruszewska@phdstud.ug.edu.pl (B.K.); michal.pieranski@phdstud.ug.edu.pl (M.K.P.); 2 Laboratory of Virus Molecular Biology, Intercollegiate Faculty of Biotechnology, University of Gdansk and Medical University of Gdansk, 80-307 Gdansk, Poland; michal.rychlowski@biotech.ug.edu.pl

**Keywords:** antimicrobials, biofilm flow system, CDC bioreactor, *Enterococcus faecium*, *Enterococcus faecalis*, fullerene, photodynamic inactivation, rose bengal, synergy

## Abstract

*Enterococcus faecium* and *Enterococcus faecalis* are opportunistic pathogens that can cause a vast variety of nosocomial infections. Moreover, *E. faecium* belongs to the group of ESKAPE microbes, which are the main cause of hospital-acquired infections and are especially difficult to treat because of their resistance to many antibiotics. Antimicrobial photodynamic inactivation (aPDI) represents an alternative to overcome multidrug resistance problems. This process requires the simultaneous presence of oxygen, visible light, and photosensitizing compounds. In this work, aPDI was used to resensitize *Enterococcus* spp. isolates to antibiotics. Antibiotic susceptibility testing according to European Committee on Antimicrobial Susceptibility Testing (EUCAST) recommendations was combined with synergy testing methods recommended by the American Society for Microbiology. Two clinical isolates, *E. faecalis* and *E. faecium*, were treated with a combination of aPDI utilizing rose bengal (RB) or fullerene (FL) derivative as photosensitizers, antimicrobial blue light (aBL), and 10 recommended antibiotics. aPDI appeared to significantly impact the survival rate of both isolates, while aBL had no significant effect. The synergy testing results differed between strains and utilized methods. Synergy was observed for RB aPDI in combination with gentamycin, ciprofloxacin and daptomycin against *E. faecalis*. For *E. faecium,* synergy was observed between RB aPDI and gentamycin or ciprofloxacin, while for RB aPDI with vancomycin or daptomycin, antagonism was observed. A combination of FL aPDI gives a synergistic effect against *E. faecalis* only with imipenem. Postantibiotic effect tests for *E. faecium* demonstrated that this isolate exposed to aPDI in combination with gentamycin, streptomycin, tigecycline, doxycycline, or daptomycin exhibits delayed growth in comparison to untreated bacteria. The results of synergy testing confirmed the effectiveness of aPDI in resensitization of the bacteria to antibiotics, which presents great potential in the treatment of infections caused by multidrug-resistant strains.

## 1. Introduction

Most Enterococci cause a vast variety of nosocomial infections of soft tissues, abscesses, urinary tract infections or even endocarditis, which overall are caused by *E. faecalis* and *E. faecium* [1]. *E. faecalis* is known as an etiological agent of opportunistic infections including bacteremia, endocarditis, meningitis, and urinary tract and bloodstream infections [2]. *Enterococcus* species, especially *E. faecalis*, are also associated with persistent endodontic infections. The most important antibiotics against which these microorganisms express resistance are β-lactams (penicillin), cephalosporines, lincosamides, streptogramins, and aminoglycosides, whereas they can also acquire resistance to glycopeptides (e.g., vancomycin, VAN) or macrolides. The first occurrence of resistance to VAN was observed in 1980, and to date, this resistance has spread massively among *E. faecium* isolates. This urgent problem of resistance is associated with an increasing number of nosocomial infections linked with VAN-resistant *E. faecium*. These reasons explain why this organism belongs to the group of ESKAPE microbes, which are the main cause of hospital-acquired infections and are especially difficult to treat because of their resistance to many antibiotics [3]. It is worth mentioning that planktonic cultures possess a drug resistance 100 to 1000 times lower than that of biofilms, and still increasing the antimicrobial resistance crisis is an additional force to find new alternatives to currently used bactericidal methods [4,5,6]. Moreover, increasing tolerance of hospital-acquired *E. faecium* strains to handwash alcohols is another problem that requires additional procedures to prevent transmission of this pathogen in the hospital setting [7]. Antimicrobial photodynamic inactivation (aPDI) seems to match perfectly as a potential candidate method for bactericidal action against planktonic and biofilm cultures. The method requires visible light, oxygen, and a photosensitizer (PS) [8]. Absorption of photons by photosensitizing agents leads to the formation of excited states of such compounds, which through further photochemical reactions lead to the production of highly toxic reactive oxygen species (ROS) or singlet oxygen [9,10]. Such products of photooxygenation can interact with DNA, lipids and proteins, leading to cell death. The appropriate degree of photoinactivation can act as a ‘tool’ for sensitization of microorganisms to antimicrobials, which was demonstrated in a previous paper published by our team for *Acinetobacter baumannii* [11]. In the current research, exogenous PSs (rose bengal (RB) and fullerene (FL) derivative) with visible green light were used as tools for the ‘sensitization’ of *Enterococcus* clinical isolates to routinely used antibiotics.

## 2. Results

### 2.1. aPDI Significantly Influences the Survival Rate of Planktonic Cultures of Enterococcus Species

**Figure 1:**
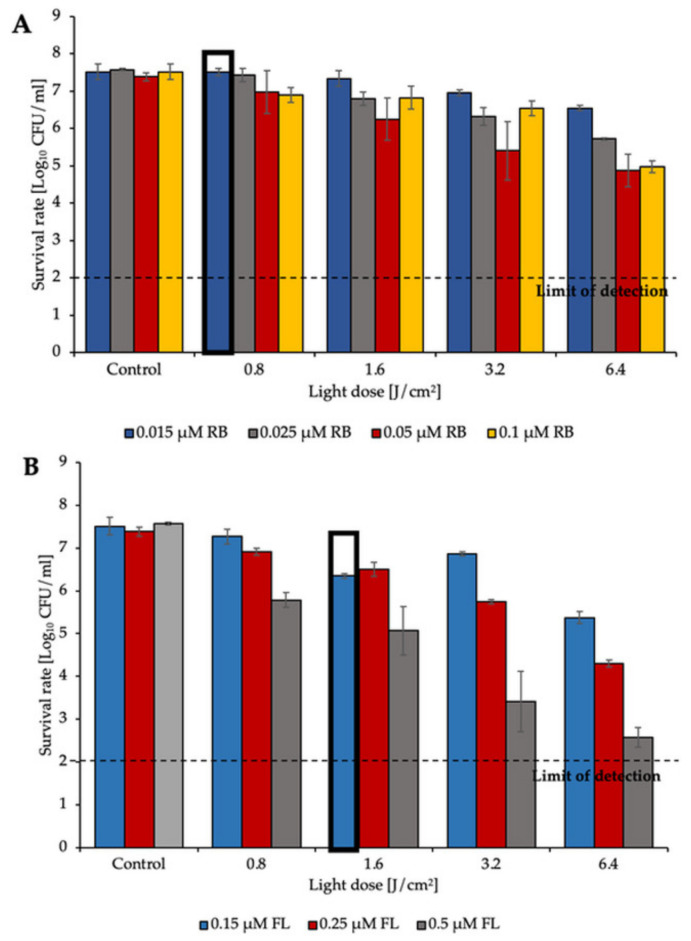
aPDI inactivation of *E. faecium* with various doses of green light and (**A**) RB concentrations (0.015, 0.025, 0.05, and 0.1 μM) or (**B**) FL concentrations (0.15, 0.25, and 0.5 μM). The experiment was performed in three biological replicates. The detection limit was 100 CFU/mL. Bold frames indicate sublethal treatment.

**Figure 2:**
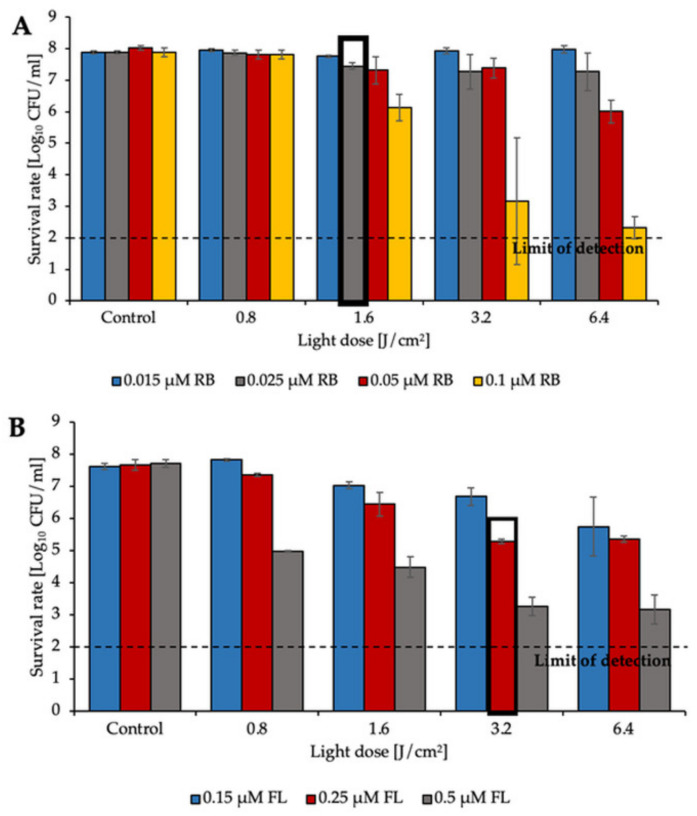
aPDI inactivation of *E. faecalis* with various doses of green light and (**A**) RB concentrations (0.015, 0.025, 0.05, and 0.1 μM) or (**B**) FL concentrations (0.15, 0.25, and 0.5 μM). The experiment was performed in three biological replicates. The detection limit was 100 CFU/mL. Bold frames indicate sublethal treatment.

The application of green light with RB revealed that the PS even at very low concentrations (0.1 μM) with a dose of green light irradiation (6.4 J/cm^2^) was able to reduce *E. faecium* viability by approx. 2.5 log_10_ CFU/mL (Figure 1A). The second PS, fullerene (FL), was administered at different concentrations (ranging from 0.15 μM to 0.5 μM) and to obtain a 5 log_10_ reduction with the same light dose (6.4 J/cm^2^) it required 0.5 μM concentration. Similar results were obtained for the second isolate (*E. faecalis*) when RB was present at a concentration of 0.1 μM, and the highest reduction (approx. 6 log_10_ CFU/mL) was detected after the application of 6.4 J/cm^2^ of green light (Figure 2A). For FL, the highest reduction was obtained when the PS was applied at concentrations of 0.5 μM. After administration of a 6.4 J/cm^2^ light dose, the reduction was estimated to be approx. 5 log_10_ CFU/mL (Figure 1B and Figure 2B).

The results presented above clearly indicate that both RB and FL based aPDI may lead to effective inactivation of two tested *Enterococcus* species. For further experiments sublethal treatments marked with bold frames were used.

### 2.2. Identification of MIC of Treatments

Adequate synergy testing required the preliminary characteristics of the studied *E. faecium* and *E. faecalis* regarding their antibiotic resistance profiles and their response to aPDI treatment. Applied techniques have indicated that both clinical isolates are multidrug-resistant pathogens; therefore, they are resistant to multiple antimicrobial agents (e.g., STR, AMP, DAP) [12], covering all possible drug categories and all mechanisms of action. Detailed characteristics are presented in Table 1.

For all of the antibiotics as well as for the photoinactivation conditions, the MIC values for both tested clinical isolates were determined. In the next set of experiments, the MIC values were used to evaluate the synergy between tested monotreatments, which was performed with the recommended methods for synergy testing (e.g., antimicrobial susceptibility testing, checkerboard assay, time-kill assay).

### 2.3. Diffusion-Based Assays Confirm aPDI/Antimicrobial Synergy

The results indicated that in the case of both phototreatments, the employment of sublethal aPDI conditions influenced the susceptibility to numerous routinely used antimicrobials, resulting in larger growth inhibition zones (in the case of the disk diffusion assay) and decreased MICs (for the E-test). The results regarding synergy testing with diffusion methods are presented in Table 2. The disk diffusion assay revealed that after treatment with aPDI, *E. faecalis* became more sensitive to STR and TGC; thus, the zones of inhibition increased by greater than or equal to 2 mm, whereas the MIC values from the E-test decreased by a minimum of 2-fold in comparison to the control, confirming the synergistic effect between aPDI and antibiotics. aPDI treatment also influenced changes in susceptibility to DOX (e.g., the inhibition zone increased from 9.4 mm to 11.3 mm) and to IPM and AMP (an increase in the inhibition zone was detected, whereas the MIC values from the E-test remained unchanged). In contrast, *E. faecium* did not respond in a similar manner to aPDI treatment. Synergy was observed for aPDI (RB) treatment with GEN (the MIC value decreased from 6 to 3 μg/mL) and TGC (the inhibition zone increased from 28.5 mm to 32.6 mm). For aPDI (FL) treatment, synergy was indicated only for DOX based on a reduction in the MIC value for the E-test from 32 to 16 μg/mL. Differences resulting from the obtained results indicate the necessity of applying multiple approaches for synergy testing; thus, one method is not sufficient to confirm the research assumptions. In addition, as light alone treatment (with no PS administration) as well as PS alone (with no light excitation) exerted no change in microbial antibiotic susceptibility, these control conditions were not included within the Table 2.

### 2.4. Serial Dilution Methods Demonstrate aPDI/Antimicrobial Synergy

The checkerboard assay method indicated that aPDI (RB) has a synergistic effect with GEN, CIP, and DAP. This conclusion was based on the FICI, the value of which was estimated as 0.38, 0.38, and 0.16 for GEN, CIP, and DAP, respectively, for *E. faecalis*. aPDI (FL) indicated synergy only with IMP (FICI = 0.25). For *E. faecium*, it was observed that aPDI (RB) has an antagonistic effect when combined with VAN/DAP. The FICI value was 8.5 and 5.25 for VAN and DAP, respectively, whereas for combined treatment with CIP and GEN, it was estimated to be 0.5, indicating synergy with aPDI (RB). A similar conclusion for *E. faecium* could also be drawn for aPDI (FL) combined with LZD. All results from the checkerboard assay are presented in Table 3. In addition, as light alone treatment (with no PS administration) as well as PS alone (with no light excitation) exerted no change in microbial antibiotic susceptibility, these control conditions were not included within the Table 3.

### 2.5. Time–Kill Curve Assay Confirms aPDI/Antimicrobial Synergy

**Figure 3:**
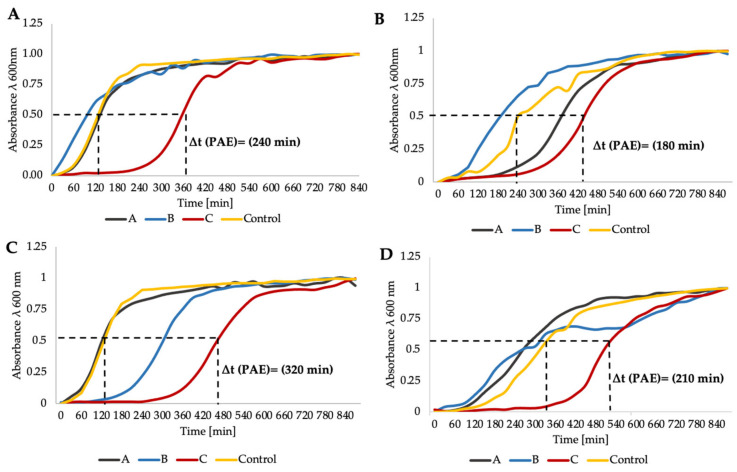
Postantibiotic effect testing. (**A**) Growth curve analysis of aPDI (FL)/GEN combined treatment for *E. faecalis*; (**B**) Growth curve analysis of aPDI (FL)/LZD treatment for *E. faecium*; (**C**) Growth curve analysis of aPDI (RB)/DOX treatment for *E. faecalis*; (**D**) Growth curve analysis of aPDI (RB)/DAP treatment for *E. faecium*. Phototreatments (aPDI (RB)/(FL)) were employed with 1/2 MIC doses and are presented on graphs with symbols (A). Antibiotics (LZD, DAP, GEN, and DOX) were administered at the MIC and are represented in the figure by symbol B. The combination of light and antibiotics is presented as symbol C (1/2MIC aPDI + MIC antibiotic). Only one representative curve is presented.

The time–kill assay, i.e., post antibiotic effect (PAE), represented another method to investigate the synergy or other interactions between aPDI (RB)/aPDI (FL) and antibiotics. For both *E. faecium* and *E. faecalis*, a synergistic effect was observed for all of the tested antibiotics (with the exception of AMP and Q-D) when combined with aPDI (FL). A characteristic “shift” of the growth curve was detected both for FL and RB aPDI and most of antibiotics; however, only four representatives were used for visualization, i.e., aPDI (FL)/GEN (Figure 3A), aPDI (FL)/LZD (Figure 3B), aPDI (RB)/DOX (Figure 3C) and aPDI (RB)/DAP (Figure 3D). In addition, as light alone treatment (with no PS administration) as well as PS alone (with no light excitation) exerted no change in microbial antibiotic susceptibility, these control conditions were not included within the Figure 3.

### 2.6. aPDI/Antimicrobials Exerts Numerous Synergies

All of the tests regarding synergy testing between antibiotics and aPDI revealed that for *E. faecium*, the prevalence of synergy was indicated for GEN with aPDI (RB) and for DOX combined with aPDI (FL) (Table 4). *E. faecalis* responded better to photoinactivation, which was reflected in the increased number of observed synergies between aPDI and antimicrobials. For example, after application of aPDI, increased susceptibility was indicated for two antibiotics, namely, STR and TGC, which was confirmed with multiple methods (Table 5).

### 2.7. aPDI/Antimicrobial Synergy Can Be Reached in the Mature Biofilm Model

**Figure 4:**
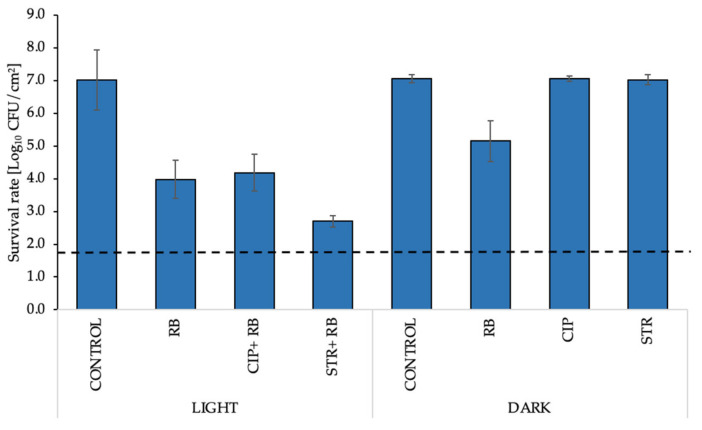
Assessment of the survival rate of *E. faecalis* biofilms grown on coupons and treated under various conditions: control (nontreated); RB (5 μM); CIP (5× MIC); STR (3× MIC) in dark or treated with green light (7.95 J/cm^2^). For each condition, three coupons were analyzed. The detection limit was 39.5 CFU/cm^2^.

**Figure 5:**
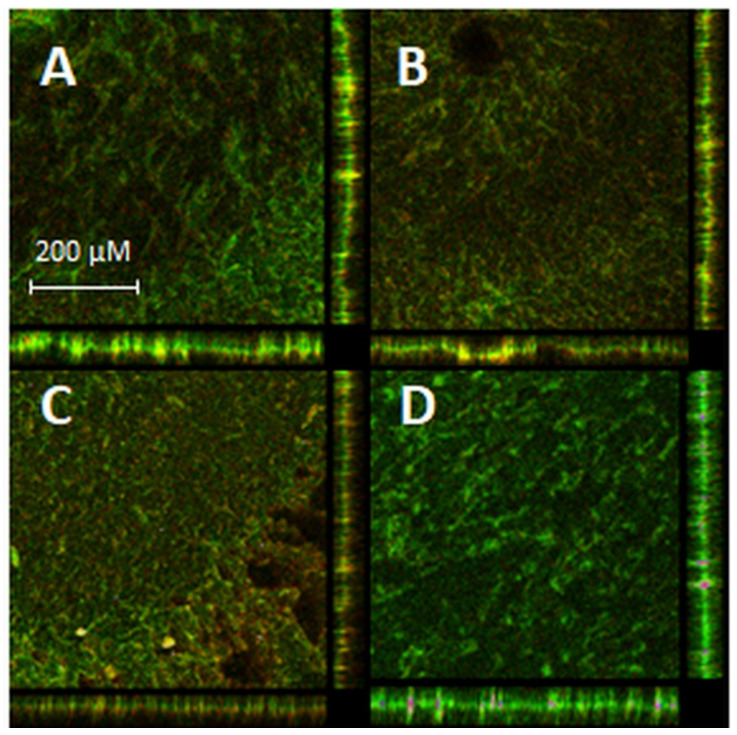
CLSM assessment of *E. faecalis*-treated biofilms. Biofilms were grown for 24 h on coupons, exposed to RB (5 μM) for 15 min and irradiated twice with a dose of green light at 7.95 J/cm^2^. Biofilms were stained with the BacLight Live/Dead kit. Panel (**A**): biofilm exposed to aPDI (RB); panel (**B**): aPDI (RB)-treated biofilm exposed to 5× MIC of CIP; panel (**C**): aPDI (RB)-treated biofilm exposed to 3× MIC of STR; panel (**D**): control (nontreated biofilm).

For the experimental procedures, RB was applied at a 10-fold higher concentration (5 μM) than that in the planktonic culture, whereas STR and CIP were applied at concentrations of 3× MIC and 5× MIC, respectively. Coupons with biofilms were irradiated twice from each side with a dose of green light of 7.95 J/cm^2^. Increased concentrations of all compounds are associated with a higher resistance of biofilm cultures to the treatment conditions. aPDI of *E. faecalis* biofilm culture with RB reduced the bacterial viability by 3.1 log_10_ CFU/cm^2^, and when combined with 3× MIC of STR, the reduction increased to 4.4 log_10_ CFU/cm^2^. The addition of 5× MIC of CIP with a PS reduced the viable cell count by 2.9 log_10_ CFU/cm^2^ (Figure 4). The results estimated by CFU/cm^2^ counting were confirmed by confocal laser scanning microscopy (CLSM) images of stained biofilm cells before and after mono- and combined aPDI therapy. The images of coupons (Figure 5A–D) with biofilms revealed that the combination of aPDI (RB) with CIP (Figure 5B) or STR (Figure 5C) led to an increased presence of red fluorescent cells, which indicated biofilm damage upon treatment.

This is the first report of a resensitization of cells growing as a mature biofilm to antibiotic treatment upon photoinactivation. These important results were confirmed by CFU/cm^2^ determination and confocal microscopy analysis. We were able to observe the bactericidal effect (approx. 4 log_10_ CFU/cm^2^ viability reduction) of the aPDI (RB) and STR combination on biofilm cells.

### 2.8. Increased ROS Generation Can Explain the Mechanism Underlying the Observed Synergies

**Figure 6:**
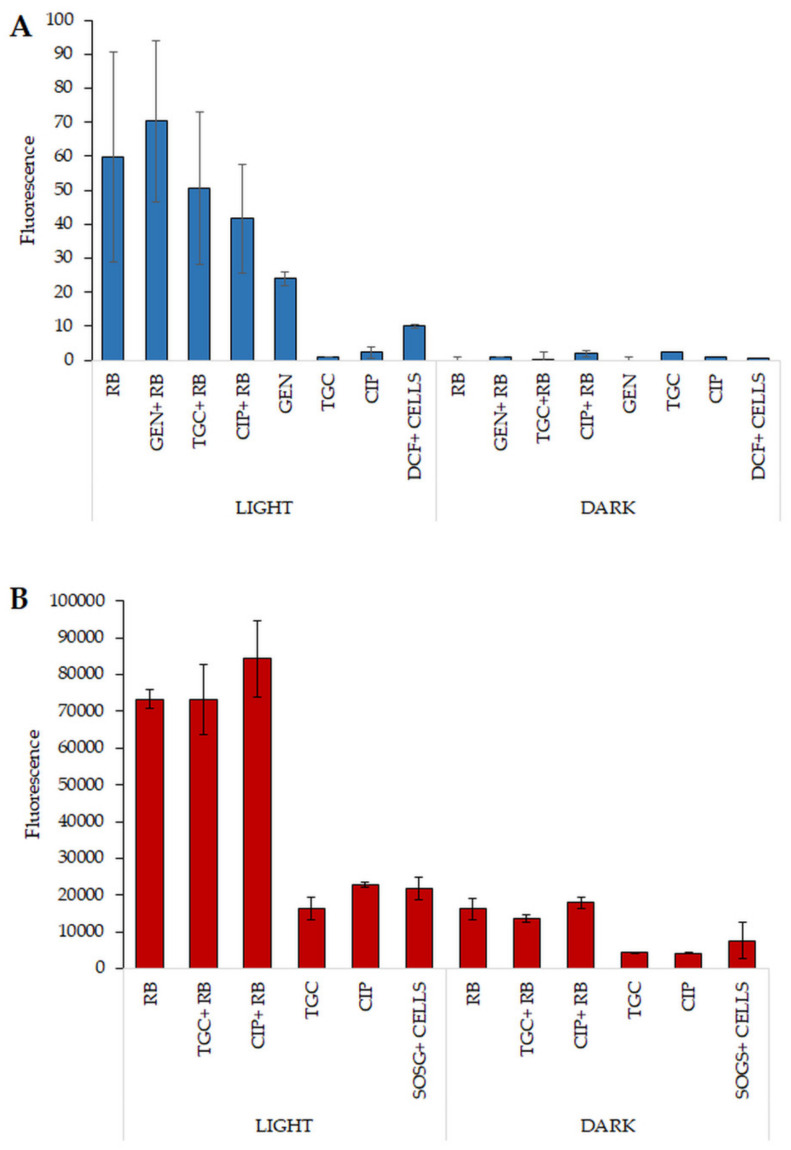
Reactive oxygen species and singlet oxygen identification. (**A**) Exposure of *E. faecalis* to various oxidative stress conditions and antibiotic monotherapies at MIC concentrations. For experimental purposes, dark controls of the tested combinations were also analyzed. The fluorescence of DCF was observed using wavelengths of 521 nm (emission) and 488 nm (excitation) with an EnVision multilabel plate reader (PerkinElmer, Waltham, MA, USA). The values are the mean of three independent experiments. (**B**) Cell suspensions of *E. faecalis* were exposed to mono- and combination therapies to detect singlet oxygen production. Fluorescence was measured at excitation/emission wavelengths of 505/523 nm with an EnVision multilabel plate reader (PerkinElmer).

To investigate whether combinations of antibiotics and photoinactivation can lead to increased production of ROS as well as singlet oxygen, various fluorescent probes were used. Application of various fluorescent probes, i.e., dichlorofluorescein (DCF) and 3′-(*p*-Aminophenyl) fluorescein (APF), is associated with different fluorescence responses of these compounds to ROS. Specific ROS (e.g., hydroxyl radicals) lead to different levels of fluorescence for each probe. From the literature data, it is well known that many antibiotics can exert their bactericidal activity due to stimulation of ROS formation [13]. To investigate whether this phenomenon could also be observed for combined aPDI/antimicrobial treatment, combinations of antibiotics—such as TGC, GEN, and CIP (at MIC concentrations)—with aPDI at dose of MIC were tested. After exposure of bacterial cells to the tested antibiotics and MIC dose of aPDI (RB), increased production of ROS was detected only for GEN. For CIP and TGC, exposure to the combined treatment did not reveal the additional production of ROS (Figure 6A). The observed effect could explain the synergy between GEN and aPDI (RB) in an in vitro model of *E. faecalis* eradication. Another fluorescent probe that was used in the experiment is also strictly associated with the production of various ROS. APF was tested with the same antimicrobials as described above under the same experimental conditions. The results of this experiment did not confirm any increased ROS production upon treatment with aPDI (RB) and GEN, CIP, or TGC (data not shown). For the detection of singlet oxygen, a SOSG probe was used to test the synergy between TGC or CIP and aPDI (Figure 6B). This experiment revealed increased singlet oxygen production (represented by the highest fluorescence level) after exposure to the combination of aPDI (RB) and CIP. The signal was higher than that with both aPDI (RB) monotherapy and the combination of aPDI (RB) and TGC. For the three different probes, increased ROS and singlet oxygen production was confirmed for the two different antimicrobials, indicating that increased levels of ROS and/or singlet oxygen may be responsible for the synergistic effect of the combined treatment.

### 2.9. Increased Permeabilization Could Explain the Mechanism of the Observed Synergies

**Figure 7:**
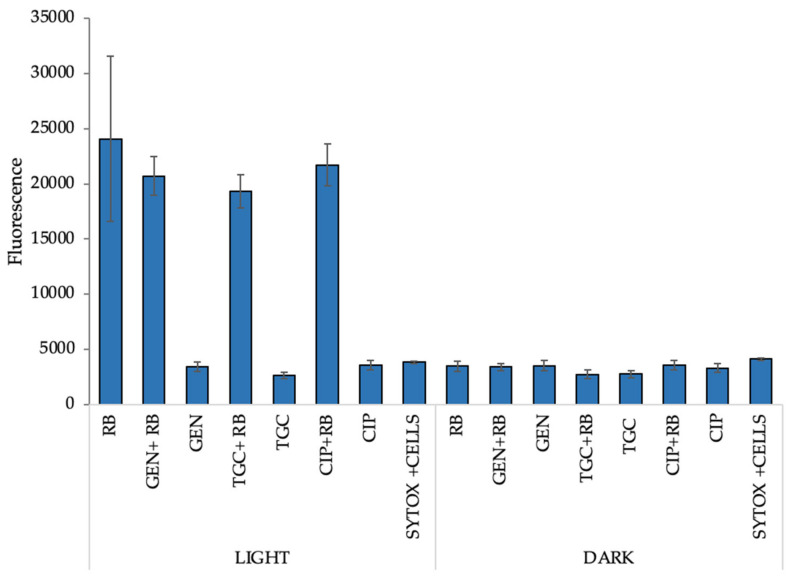
*E. faecalis* cell membrane integrity. Samples were treated with aPDI and aPDI combined with antibiotics at MIC concentrations and exposed to the SYTOX Green label. Additionally, the control for cells and labels was prepared (SYTOX + cells). The absorbance was measured with an EnVision multiplate reader (PerkinElmer) with 504/523 nm excitation/emission filters. The experiment was performed in three independent biological replicates.

To investigate whether aPDI (RB) can lead to permeabilization of the bacterial membrane, SYTOX Green was used. Increased permeabilization could result in more efficient antibiotic penetration into bacterial cells, leading to increased damage and cell death, thus explaining the phenomenon of synergy. For this purpose, SYTOX Green was used as a high-affinity nucleic acid compound that can interact with intracellular DNA [14]. The leakage of DNA is a result of the cell permeabilization process upon photoinactivation. When aPDI (RB) was applied, the most severe damage to the cell membrane was observed under this condition (Figure 7); thus, we are convinced that the increased permeabilization may be the most important reason for the observed synergistic effect between antimicrobials and aPDI as it may result in increased antibiotic uptake.

## 3. Discussion

Disturbance of oral human microflora can rapidly influence the growth and spread of nosocomial pathogens—e.g., *E. faecium*, *Streptococcus mutans*, *Porphyromonas ginvigalis*, and *Lactobacillus gasserii*—leading to the development of intraoral diseases. It is worth mentioning here that *E. faecalis* is commonly detected in persistent infections after failed endodontic treatments, and *E. faecium* is mainly associated with infections caused by the use of indwelling medical devices, e.g., central venous and urinary catheters [15,16]. Before the era of widespread application of antibiotics, most bacterial infections were fatal for patients. The discovery of the bactericidal or bacteriostatic activity of some compounds was shown to be a very effective therapeutic solution. Since then, antibiotics have been used to treat infections caused by many types of bacteria. However, there has now been an increase in the incidence of diseases caused by microbes resistant to many types of therapeutics and a decline in the number of new antibiotics introduced. Hence, this kind of therapy will be ineffective in the future. Antimicrobial photoinactivation of bacteria (aPDI) is a promising approach, but it also has a few limitations (e.g., depth of penetration of light); regardless, many positive applications and evidence of success have been observed. Photoinactivation is often presented as a method in the treatment of peri-implantitis, tooth canal infections, and other oral infections [17,18].

The first case of the significant potential of aPDI in sensitizing *Enterococcus* spp. strains resistant to VAN appeared in the literature in 2013 [19]. This study presented an in vivo model of larval infection of *Galleria mellonella* with *E. faecium*. The application of VAN with light and methylene blue (MB) increased the survival rate of infected caterpillars in comparison to treatment with only aPDI or VAN alone. Another example of successful application of aPDI against this microorganism was described by Kang et al. in 2019. Light treatment of *E. faecium* planktonic culture in the presence of curcumin and protoporphyrin IX significantly reduced bacterial growth [20]. Moreover, it is well known that biofilm cultures are more resistant to bactericidal treatments than planktonic cultures due to the presence of a matrix that consists of polysaccharides, proteins, and nucleic acids, which constitute a mechanical barrier for antimicrobial compounds. Nevertheless, the results published by López-Jiménez et al. showed that eradication of biofilms is still possible. In their experiments, MB or toluidine blue O (TBO) excited with 670 or 628 nm wavelength light led to severe damage to biofilm cells and even increased the roughness of the biofilm surface [21].

The second representative of the genus *Enterococcus*, *E. faecalis*, was also eradicated by phototreatment of the biofilm cultures. For example, it was proven that aPDI can simultaneously affect biofilms via damage to bacterial cells and the extracellular matrix. Photoinactivation with MB was reported to reduce the *E. faecalis* biofilm surface by 89% in comparison to the samples incubated only with the PS. In multispecies biofilms (*E. faecalis* and *P. aeruginosa*), aPDI with MB reduced the biofilm-covered area by 59.3% [22]. Moreover, eradication of *E. faecalis* in the root canal was shown to be possible with the application of MB with red light (660 nm) [23]. The potentiation of the antimicrobial efficacy of RB and green light was proved by experiments performed by Li et al. The addition of potassium iodide (KI) (at a concentration of 100 mM) increased the effectiveness of the reduction in planktonic culture with aPDI by an additional reduction of 4 log_10_ CFU/mL. The same effect was observed when biofilm cells were treated with RB aPDI. Moreover, Shrestha et al. described the efficacy of RB-conjugated chitosan, used as a PS, which led to eradication of planktonic culture of *E. faecalis* and reduced the bacterial viability count in biofilms by approx. 3 log_10_ CFU/cm^2^ [24]. These experiments confirmed that the effectiveness of RB as well as MB at very low molar concentrations against this pathogen can be potentiated.

In the current study, the differences in the response of both isolates to various PSs were demonstrated. *Enterococcus* spp. show greater sensitivity to RB than to FL. This finding may be related to the mechanism of action of both PSs. In the case of FL, it has been described that apart from the production of singlet oxygen in polar solvents, an important mode of action of this PS is the permeabilization of cell membranes. Research conducted by our team has shown that FL accumulates mainly in cell sheaths [14]. However, the mechanism of action of RB is mainly related to the production of singlet oxygen. In subsequent studies, the ability of RB to attach to the cell membranes of *E. faecalis* was demonstrated by flow cytometry [25], which may potentially explain the greater effectiveness of RB than FL against *Enterococcus* spp. The results of our experiments highlight the effectiveness of aPDI with RB or FL against two multidrug-resistant (MDR) isolates: *E. faecalis* and *E. faecium*. A high level of resistance was observed against antibiotics such as STR, DAP, and AMP, which was reduced after aPDI treatment, especially in the case of *E. faecalis*. Synergy testing between aPDI and antimicrobials was performed with multiple methods regarding the data presented in our published review paper [26]. The resistance to STR of *E. faecalis* isolate was reduced after application of aPDI (RB and FL) (the inhibition zone increased by 2.9 mm). Additionally, after application of STR with the aPDI (RB and FL) combination, a delay in bacterial growth was detected. The checkerboard assay is an excellent method to investigate the combinations of two factors; however, this method revealed synergy or even antagonism between aPDI and antimicrobials for only a few combinations. GEN and CIP exhibited synergistic effects with aPDI (RB) when applied against both *Enterococcus* species. Individual synergy in the case of *E. faecium* occurred for antibiotics DAP, IPM, or LZD with aPDI (RB and FL), and antagonism was revealed for DAP and VAN when combined with aPDI with FL. Moreover, the PAE results revealed that bacterial growth can be significantly disturbed after combined treatment application in comparison to monotherapies. For most of the combinations, the PAE was positive or partially positive. It is also worth mentioning that for each photoinactivation treatment, regarding the presence of RB and FL, MICs were determined for both strains and PSs. The concentrations or treatment doses presented in Table 1 could not be used in experiments regarding the PAE. Such applied doses of aPDI with FL were too harsh for bacterial cells, and the regrowth effect could not have been observed. Therefore, for synergistic effect determination and the ability to observe the effect of aPDI with antimicrobials in terms of MIC values, the estimated photoinactivation conditions had to be weakened. Despite the very high resistance of the *E. faecalis* isolate to STR, resensitization and synergy with aPDI (RB) were confirmed for planktonic culture and biofilm cells. The combined treatment successfully reduced the bacterial load for biofilm culture from 7.1 to 2.7 CFU/cm^2^. One could ask whether the sequence of treatments, i.e., starting with aPDI or antimicrobials, may affect the results. The sequence treatment studied within the current work included the application of aPDI as a first step of experimental procedure, nevertheless, the alternative sequence has also been studied (data not shown). The performed analysis revealed that similar synergies could be demonstrated regardless the sequence used. Obviously, when studying tetracyclines, that could also serve as standard PSs and be excited with appropriate wavelength irradiation, one could assume that starting with antibiotic application followed with light treatment should enhance the bactericidal outcome, nevertheless, using our experimental conditions, the expected increase in killing efficacy was not observed (data now shown). To investigate the mechanism of the obtained synergy, multiple fluorescent probes were used to detect the potentially increased production of singlet oxygen or other ROS. DCF revealed increased radical production in combination with aPDI (RB) and GEN, but the fluorescence level was quite low when compared with that of the APF probe. The second indicator (APF) confirmed a high fluorescence level for all tested antimicrobials when combined with aPDI (RB); however, this level was slightly lower than that for the monotherapy (aPDI RB); thus, the APF results did not confirm the increased production of ROS in the combined treatment. SOSG, which is suited to the detection of singlet oxygen, confirmed increased production of this radical when aPDI (RB) was combined with CIP. The last experiment trying to explain the occurrence of synergy employed the intracellular DNA probe SYTOX Green. This compound efficiently binds to nucleic acids after they leak out of cells through the permeabilized membrane. aPDI treatment leads to increased permeabilization of the cells which may be the most important reason of observed synergy. The increased membrane permeabilization may result in increased antibiotic uptake and lead to enhanced killing efficacy.

Despite demonstrating that aPDI leads to significant membrane permeabilization which could partially explain the observed synergy, the mechanism of synergistic effect remains poorly understood. Resensitization of microbes to a particular antibiotics after exposure to sub-lethal aPDI could primarily result from the following reasons: (i) aPDI inactivation of the microbial agents responsible for drug resistance mechanisms; (ii) aPDI caused increased cell envelopes permeabilization leading to increased diffusion of antibiotic into the microbial cell; (iii) aPDI mediated disruption of membrane components leading to the change in membrane potential which may further affect PS uptake or its binding to cell envelope; and (iv) increased ROS production resulting from antimicrobial ROS generation.

aPDI leads to inactivation of multiple cellular components, i.e., proteins, lipids or genetic material, thus, it exerts deleterious effects against numerous virulence factors and enzymes responsible for antimicrobial resistance mechanisms. *Enterococcus* spp. display a variety of enzymes and proteins being key factors of drug resistance mechanisms, i.e., acetyl-, phospho-, and adenyltransferases, transpeptidases, or proteins building efflux pumps [27,28,29,30]. Possible aPDI mediated inactivation of these factors could result in microbial resensitization to particular antibiotics. In case of increased membrane permeabilization, the current study provides clear evidence supporting this thesis, and indeed, this aPDI caused membrane permeabilization could be the most important reason for observed synergistic effect. Finally, we hypothesize that aPDI may lead to the disruption of cell envelope components affecting membrane potential, i.e., lipoteichoic acid (LTA) present in Gram-positive microbes. It has been evidenced that inactivation of LTA may lead to significant increase in antibiotic diffusion resulting in enhanced killing efficacy of antibiotic treatment [31]. In addition, numerous studies demonstrate that antibiotic lethality is accompanied by ROS generation [31,32,33]; thus, the overall oxidative stress could be significantly enhanced when combined aPDI/antimicrobial treatment is applied. This effect could also be the reason of the observed synergistic effect.

The most intriguing aspect of the observed synergy is providing explanation why the synergy could be demonstrated only for few antibiotics and what factors determine that specific antimicrobials may exert its increased efficacy upon sub-lethal aPDI treatment. Nevertheless, this explanation is still being undiscovered and worthy further investigations. We have made an effort to identify some chemical features of tested antimicrobials regarding its molecular weight, polar surface area, formal and physiological charge, complexity, water solubility, pKa, or mechanism of action that could potentially group studied antibiotics according their synergistic cooperation with aPDI; however, none of tested feature was demonstrated to be corelated with the observed synergy.

The results of the synergy testing experiments confirm the effectiveness of aPDI in sensitizing bacteria to antibiotics. This modality holds great potential for treating infections caused by multidrug-resistant strains that are mainly acquired in hospitals. A great advantage of aPDI is the nonspecific mechanism of action allowing comprehensive cell destruction. This approach prevents bacteria from developing resistance against this type of treatment, representing a significant advantage of aPDI treatment despite the risk of increased tolerance development, as presented by our team in two recently published articles [34,35]. However, the results of these studies may be clinically applicable, especially in the fields of dentistry or wound management. The ability of biofilm eradication in combined treatment, as presented here, is of great importance and indicates that this method is efficient despite obvious limitations.

## 4. Materials and Methods

### 4.1. Bacterial Strains and Culture Conditions

In this study there were two clinical isolates used: *E. faecium* EU87 and *E. faecalis* EU92. Strains were kindly provided with dr Valentina Ebani (Pisa, Italy). Tryptic Soy Broth (bioMérieux, Craponne, France) with 1.5% agar (BTL, Warsaw, Poland) plates were used for colony forming unit (CFU) enumeration and tryptic soy broth (TSB) (bioMérieux, Craponne, France) was used for overnight planktonic cultures and batch and flow phase of biofilm culture.

### 4.2. Photosensitizers

4,5,6,7-Tetrachloro-2′,4′,5′,7′-tetraiodofluorescein disodium salt (RB) powder was purchased from Sigma Aldrich (Munich, Germany). The stock solution was prepared in double-distilled water (ddH_2_O) and kept in the dark at 4 °C. Fullerenopyrrolidine (N-methylpyrrolidinium fullerene iodide salt) was purchased from ProChimia (Sopot, Poland). A stock solution of the compound was prepared in dimethylsulfoxide (DMSO)/ddH_2_O solution (1:9, *v*/*v*) and kept in the dark at 4 °C.

### 4.3. Antibiotics

Gentamycin (GEN), doxycycline (DOX), streptomycin (STR), ciprofloxacin (CIP), imipenem (IPM), vancomycin (VAN), and ampicillin (AMP) were purchased from Sigma Aldrich. Daptomycin (DAP), linezolid (LZD), and tigecycline (TGC) were purchased from Cayman Chemicals (Ann Arbor, MI, USA). Stock solutions at concentrations of 10 mg/mL were prepared in the recommended solvent and stored at −20 °C.

### 4.4. Light Sources

The custom constructed LED-based light source was used: emitting λ_max_ 522 nm light with a radiosity of 10.6 mW/cm^2^ (FWDH (full width half maximum) 34 nm) (Cezos, Gdynia, Poland).

### 4.5. Photodynamic Inactivation of Planktonic Cultures

Overnight culture (1 colony transferred into 5 mL of tryptic soy broth (TSB) and incubated for 18 h at 37 °C with shaking at 150 rpm) of *E. faecium* and *E. faecalis* were adjusted to 0.5 McFarland (McF) units (Densi-La-Meter II, ERBA) in phosphate-buffered saline (PBS) (Sigma Aldrich, Inc, Munich, Germany), which corresponds to a cell density of approx. 10^7^ CFU/mL. Working solutions of RB were prepared in ddH_2_O or in the case of FL in a mixture of distilled water:DMSO (9:1 *v*/*v*). The bacterial suspension and PS solution were mixed and incubated in the dark at room temperature (RT) for 15 min. Then, the samples with PSs (100 μL) were illuminated. Afterwards, the samples were serially diluted in PBS and transferred onto tryptic soy agar (TSA) plates. After 18–20 h of incubation at 37 °C, colonies were counted, and the CFU/mL values were determined. Samples with RB and FL were illuminated with 522 nm light.

### 4.6. Determination of Sublethal and Lethal Doses of aPDI for Planktonic Cultures

Bacterial overnight cultures were suspended to obtain an optical density of 0.5 McF. Next, probes for the green light were mixed with PS solutions in 96-well plates and incubated for 15 min in the dark. Bacteria were irradiated with various light doses and then serially diluted, streaked on TSA plates and incubated at 37 °C for 16 h. After 16 h, colonies were counted, and the CFU/mL values were estimated. In addition, two control samples were prepared: 1, with no PS and with light to check bacterial growth; and 2, with PS and incubation in the dark to check the possible toxicity of PS. Sublethal doses (which reduce bacterial viability from 0.5 to 2 log_10_ CFU/mL) were calculated based on the survival rate of bacteria treated with aPDI in comparison to untreated bacteria. The lethal dose was determined as a ≥ 3 log_10_ CFU/mL reduction in viability.

### 4.7. Determination of MIC Doses of aPDI

Overnight cultures of both strains were diluted to obtain 0.5 McF in brain–heart infusion broth (BHI media; BioMerieux, France) and then diluted 10-fold. The experiment was not performed in Mueller-Hinton medium (MHE) due to the very weak growth of *Enterococcus* species. In the next step, samples were mixed with a solution of PS at the tested concentrations in 96-well plates. Suspensions were then incubated in the dark for 15 min and exposed to various light doses. Subsequently, the plates were incubated at 37 °C for 16–20 h, and bacterial growth was assessed optically in microtiter wells. The experiment was conducted in three independent replicates.

### 4.8. MIC Determination of Tested Antibiotics

Overnight cultures of both strains were adjusted to 0.5 McF in BHI and then diluted 10-fold. Next, probes were administered with antibiotics to reach the tested range of concentrations (from 1024 to 0.03125 μg/mL) in 96-well plates. Afterwards, the plates were incubated at 37 °C for 16–20 h. Bacterial growth was assessed optically in microtiter wells. The experiment was conducted in three independent replicates.

### 4.9. Synergy Testing

#### 4.9.1. Antimicrobial Susceptibility Testing (Disk Diffusion Method and E-Tests)

Overnight cultures were diluted in PBS to obtain 0.5 McF. For the light-treated probes, sublethal doses of PSs were added. Next, the probes were incubated in the dark for 15 min and then exposed to sublethal doses of light. The next steps were the same for the treated and untreated probes. Then, 15 min after preparing the 0.5 McF suspension for untreated probes or immediately after light exposure for treated probes, the suspensions were streaked on MH agar plates (MHE, BioMerieux, France). After another 15 min, E-tests and disks with the tested antibiotics were placed on the plates. After 15 min of incubation at RT, the plates were placed in an incubator for 16–20 h at 37 °C. For antibiotics in disks, a synergistic effect was identified when the difference between the untreated and treated inhibition zones was greater than or equal to 2 mm. In the case of E-tests, synergy was confirmed if the minimum inhibitory concentration (MIC) of the treated probe was at least 2-fold lower than of the untreated probes (control).

#### 4.9.2. Checkerboard Assay

Overnight cultures of both strains were diluted to obtain 0.5 McF in BHI and then diluted 10-fold. Bacterial suspensions were placed in 96-well plates combined with different concentrations of antibiotics: 2 MIC, MIC, 1/2 MIC, 1/4 MIC, 1/8 MIC, 1/16 MIC, 1/32 MIC, and 0 MIC. Next, the wells in columns were diluted 2-fold with PS to obtain final PS concentrations with MICs as follows: MIC, 1/2 MIC, 1/4 MIC, 1/8 MIC, 1/16 MIC, 1/32, 1/64 MIC, 1/128 MIC, 1/256 MIC, 1/512 MIC, and 0 MIC. All cells were incubated in the dark for 15 min and then exposed to irradiation at MIC doses. Next, the plates were incubated for 16–20 h at 37 °C. Bacterial growth was assessed, and the fractional inhibitory concentration index (FICI) coefficient was calculated (FICI = FIC_A_ + FIC_B_). FIC_A/B_ = MIC of factor A/B in combination/MIC of factor A/B alone. Synergistic effects were observed when FICI ≤ 0.5, and antagonism was observed when FICI > 4; 4 < FICI > 0.5 means no interaction.

#### 4.9.3. Postantibiotic Effect

Overnight cultures of both strains were diluted in BHI (1:20). A few combinations of agents were prepared: A, 1/2 MIC aPDI; B, MIC of antibiotic; C, 1/2 MIC of antibiotic; D, MIC of antibiotic + 1/2 MIC aPDI; and E, 1/2 MIC of antibiotic + 1/2 MIC aPDI. All probes were incubated in the dark for 2 h in an orbital incubator at 150 rpm. Next, the agents were removed by two washing steps, and bacteria were finally suspended in fresh BHI. Probes A, D, and E were exposed to irradiation in 1/2 MIC aPDI. Control samples were not exposed to any agents. Next, all samples were transferred to 96-well plates and placed in an EnVision multilabel plate reader (PerkinElmer, Waltham, MA, USA) for 16 h, which monitored the optical density (λ 600 nm) of cultures every 0.5 h. All data were normalized, and the postantibiotic effect (PAE) was calculated on the basis of the formula PAE = T – C (T, time required to reach OD_600_ = 0.5 after removal of the investigated agent; C, time required to reach OD_600_ = 0.5 of untreated bacteria). PAE ≥ 3 h indicates a synergistic effect, and 1.5 h ≤ PAE < 3 h indicates partial synergy.

### 4.10. Determination of Singlet Oxygen Production

An experiment was conducted for *E. faecalis* and RB with TGC or CIP. Overnight cultures were diluted in PBS to 0.5 McF. Additionally, 500 μM solutions of singlet oxygen sensor green probe (SOSG) purchased from Thermo Fisher Scientific (Waltham, MA, USA), was prepared according to the manufacturer’s guidelines. Bacteria were mixed with PS and antibiotics (MIC) in different combinations and transferred to black sterile 96-well plates. To 100 μL of total volume, 1 μL of SOSG solution was added to estimate the final concentration of 5 μM. Then, the probes with PS were incubated for 15 min in the dark and exposed to light at MIC and 1/2 MIC doses. Next, fluorescence was measured using an EnVision plate reader at excitation/emission wavelengths of 488/525 nm. The experiment was performed in three independent replicates.

### 4.11. Determination of Production of ROS/Radicals

3′-(*p*-Aminophenyl) fluorescein (APF) is a specific probe for hydroxyl radicals (•OH) and 2′,7′-dichlorodihydrofluorescein diacetate (DCF) is specific also for (•OH), but also for other oxygen radicals. Experiments were conducted for *E. faecalis* and RB with TGC, GEN, or CIP. Overnight cultures were diluted in PBS to 0.5 McF. Bacteria were mixed with PS and antibiotics (MIC) in different combinations and transferred to black and sterile 96-well plates. To 100 μL of full volume, 1 μL APF solution or 5 μL of DCF solution (Thermo Fisher Scientific, Waltham, MA, USA) was added. Then, probes with PS were incubated for 15 min in the dark and exposed to light at MIC and 1/2 MIC doses. Next, fluorescence was measured using an EnVision plate reader at excitation/emission wavelengths of 490/515 nm for APF and 492–495/517–527 nm for DCF. The experiment was performed in three independent replicates.

### 4.12. Cell Membrane Integrity Assay

SYTOX Green has high affinity for DNA released from cells with permeabilized membranes. An experiment was conducted for the *E. faecalis* isolate and RB aPDI with TGC, GEN, or CIP. Overnight cultures were diluted in PBS to 0.5 McF. Bacteria were mixed with PS and antibiotics (MIC) and transferred to 96-well plates. To 100 μL of full volume, 1 μL of SYTOX Green (Molecular Probes, Eugene, OR, USA) solution was added. Then, the probes with PS were incubated for 15 min in the dark and exposed to light at MIC and 1/2 MIC doses. Next, fluorescence was measured using an EnVision plate reader at excitation/emission wavelengths of 488/523 nm. The experiment was performed in three independent replicates.

### 4.13. Materials and Methods Referring to Biofilm Culture

#### 4.13.1. Biofilm Culture Conditions

**Figure 8:**
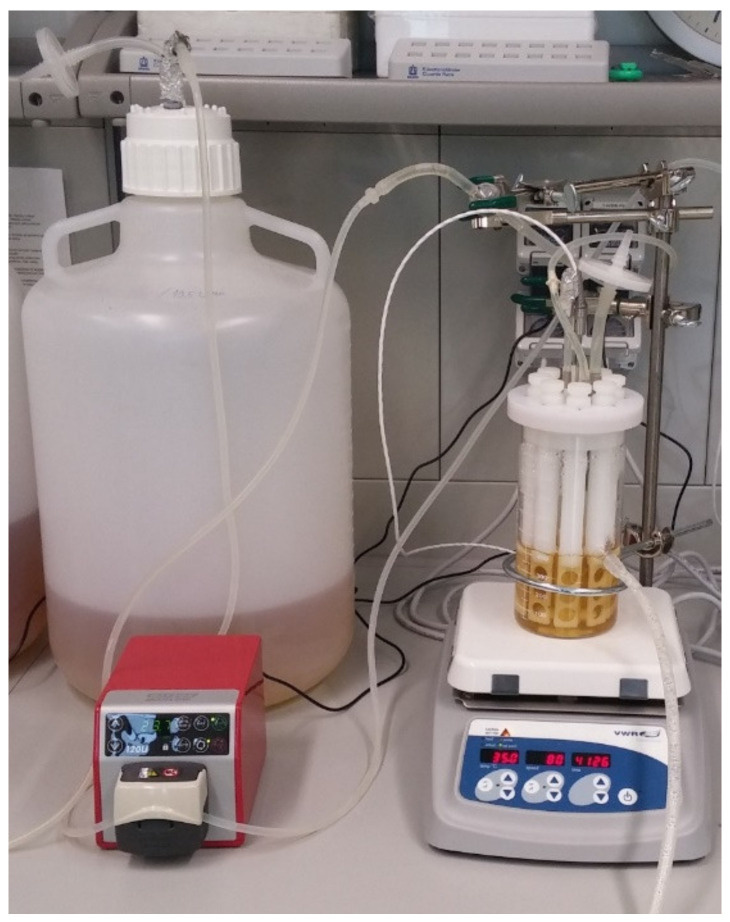
CDC biofilm reactor (BioSurface Technologies, Bozeman, MT, USA).

For biofilm culture, a CDC biofilm reactor (BioSurface Technologies, Bozeman, MT, USA), presented in Figure 8, was used with coupons made of porous polycarbonate. Before each culture, the coupons were sonicated for 10 min in 1% sodium dodecyl sulfate (SDS), washed in distilled water, sonicated for 10 min in distilled water, washed, incubated for 2 h in 2 M hydrochloric acid and finally washed in distilled water. Then, the coupons were placed in polypropylene rods, which were placed into reactors containing 500 mL of distilled water. The whole setup was autoclaved for 60 min at 10.3 psi. Water in the reactor was then replaced with 500 mL of sterile TSB (30 g/L + 100 g/L glucose) inoculated with 1 mL of 3.5 McF adjusted overnight culture of *E. faecalis*. The reactor was placed onto a magnetic stirrer with a heater set at 80 rpm and 37 °C for 24 h, referring to a batch phase. Before starting the flow phase, 1 L of 20× concentrated sterile TSB was added to a 20 L carboy containing 19 L of distilled water autoclaved for 2 h at 14.7 psi. The final concentration of broth was 30 g/L TSB with 10 g/L glucose. The carboy was connected to the reactor by silicone tubing and connected to a peristaltic pump (Watson-Marlow Fluid Technology Group, Falmouth, UK). The flow rate was set to 12.9 mL/min, and the reactor volume was 335 mL, which resulted in a residence time of 26 min, consistent with the *E. faecalis* generation time. The time of the flow phase was 24 h.

#### 4.13.2. Biofilm Treatment

Coupons with biofilm layers were incubated with RB (5 μM) and STR (3× MIC, 768 μg/mL) or CIP (5× MIC, 10 μg/mL) in PBS for 15 min and then exposed to aPDI. The coupons were irradiated for 12.5 min, turned around and irradiated again. Four control groups without irradiation were prepared: (1) with no factor; (2) only with RB in the dark; (3) with CIP; and (4) with STR. After treatment, the coupons were placed in Falcon tubes with 10 mL of PBS. Then, biofilm layers were dispersed by sonication with 40% amplitude. Each probe was sonicated for 1 min, vortexed for 1 min and incubated on ice for 1 min. The procedure was repeated three times. After this procedure, the samples were vortexed again, and 100 μL of each sample was serially diluted in PBS, streaked on TSA plates and then incubated at 37 °C for 16 h. The CFU/cm^2^ values of the coupon were calculated. The experiment was conducted in three replicates.

#### 4.13.3. Biofilm Visualization

Biofilm growth on coupons was also visualized using confocal microscopy. Visualization of biofilms was performed with a BacLight Live/Dead viability kit. Coupons without or after aPDI/antibiotic treatment were transferred to a 12-well glass-bottom plate and incubated in the presence of SYTO 9 and propidium iodide (PI) dissolved in PBS for 15 min in the dark at RT, according to the protocol described previously [36]. Specimens were imaged using a confocal laser scanning microscope (Leica SP8X) with a 10× lens (Leica, Germany). During observation, the excitation were 488 and emission wavelengths used for detecting SYTO 9 were 501–548 nm, and for detecting PI 603–649 nm. Photographs were obtained and then analyzed with Leica LAS X software.

## Figures and Tables

**Table 1:** MIC for antimicrobials and light.

Antibiotic Target	Antimicrobial Category	Antibiotic	*E. faecalis*	*E. faecium*
			MIC (μg/mL)	MIC (μg/mL)
Protein synthesis (30S)	Aminoglycosides	Gentamycin	64	32
	Tetracyclines	Doxycycline	16/8	32
	Streptomycin	Streptomycin	256 (R)	1024 (R)
	Glycylcyclines	Tigecycline	1 (R)	8/4 (R)
70S initiation complex	Oxazolidinones	Linezolid	2	1
DNA gyrase	Fluoroquinolones	Ciprofloxacin	2	2
Cell-wall synthesis	Carbapenems Glycopeptides Penicillins	Imipenem Vancomycin Ampicillin	1/0.5 1 >64 (R)	16/8 (R) 2 >1024 (R)
Cell membrane	Lipopeptides	Daptomycin	128 (R)	64 (R)
Phototherapy	aPDI (FL)	Green light + FL	28.6 J/cm^2^ + 10 μM FL *(3.6 J*/*cm^2^* *+ 0.625 μM FL)* ^1^	28.6 J/cm^2^ + 10 μM FL *(3.6 J*/*cm^2^* *+ 0.312 μM FL)*
	aPDI (RB)	Green light + RB	15.9 J/cm^2^ + 1 μM RB *(7.95 J*/*cm^2^* *+ 0.5 μM RB)*	15.9 J/cm^2^ + 1 μM RB *(15.9 J*/*cm^2^* *+ 0.5 μM RB)*

^1^ Italic font indicates the sublethal conditions used for post antibiotic effect (PAE) testing.

**Table 2:** Antimicrobials MIC change upon sublethal aPDI treatments.

Antibiotic	*E. faecalis*						*E. faecium*					
	Control		aPDI (RB)		aPDI (FL)		Control		aPDI (RB)		aPDI (FL)	
	DF ^1^	E-Test	DF	E-Test	DF	E-Test	DF	E-test	DF	E-Test	DF	E-Test
**GEN**	10 ^2^	12 ^3^	9.8	8	10.8	8	16.5	6	17.8	3	15.8	4
**STR**	11	256	**13.9**	**128 ^4^**	**15**	**128**	10	1024	10.9	≥1024	8.6	≥1024
**TGC**	22.4	19	**24.7**	**0.64**	**25.5**	≥256	28.5	0.064	32.6	0.047	26	0.64
**DOX**	9.4	32	**11.3**	**16**	10.9	**16**	13	32	10.9	32	11.6	**16**
**LZD**	24.7	2	24	1.5	25.9	2	29.6	1	31	0.75	28.7	1
**CIP**	20.5	0.75	21.8	0.5	**22.7**	0.5	22.1	0.5	22.7	0.5	21.4	0.5
**IMP**	29.3	0.75	**31.2**	0.75	29.5	0.75	10.5	32	6	32	8.9	≥32
**VAN**	13.4	2	14	2	13.8	2	21	0.5	21.1	0.38	18	0.38
**AMP**	8.4	0.5	**12.2**	0.5	**11**	0.75	6	2	6	3	6	-
**DAP**	-	1	-	1	-	1	-	2	-	1.5	-	1.5
**Q-D**	11.5	-	12.1	-	12.2	-	17.7	-	16.1	-	17.7	-

^1^ Disk diffusion; ^2^ Expressed in mm; ^3^ Expressed in μg/mL; ^4^ Bold font indicates significant change in MIC upon sublethal aPDI treatments; Abbreviations: GEN, gentamycin; STR, streptomycin; TGC, tigecycline; DOX, doxycycline; LZD, linezolid; CIP, ciprofloxacin; IMP, imipenem; VAN, vancomycin; AMP, ampicillin; DAP, daptomycin; Q-D, quinupristin-dalfopristin (Synercid); FL, fullerene; RB, rose bengal.

**Table 3:** Checkerboard FICI calculation.

Antibotic	*E. faecalis*		*E. faecium*	
	aPDI (RB)	aPDI (FL)	aPDI (RB)	aPDI (FL)
**GEN**	**0.38 ^1^**	>0.5	**0.5**	>0.5
**STR**	>0.5	>0.5	>0.5	>0.5
**TGC**	>0.5	>0.5	>0.5	>0.5
**DOX**	>0.5	>0.5	>0.5	>0.5
**LZD**	>0.5	>0.5	>0.5	**0.5**
**CIP**	**0.38**	>0.5	**0.5**	>0.5
**IMP**	>0.5	**0.25**	>0.5	>0.5
**VAN**	>0.5	>0.5	**8.5**	>0.5
**AMP**	>0.5	>0.5	>0.5	>0.5
**DAP**	**0.16**	>0.5	**5.25**	>0.5
**Q-D**	-	-	-	-

^1^ Bold indicates possible synergistic interactions; GEN, gentamycin; STR, streptomycin; TGC, tigecycline; DOX, doxycycline; LZD, linezolid; CIP, ciprofloxacin; IMP, imipenem; VAN, vancomycin; AMP, ampicillin; DAP, daptomycin; Q-D, quinupristin-dalfopristin (Synercid); FL, fullerene; RB, rose bengal.

**Table 4:** Summarized results of synergy testing for *E. faecium*.

Antibiotic	aPDI (RB)				aPDI (FL)			
	DF ^1^	E-Test	Checkerboard Assay	PAE	DF	E-Test	Checkerboard Assay	PAE
**GEN**	-	+	+	+	-	-	-	+
**STR**	-	-	-	+	-	-	-	+/-
**TGC**	+	-	-	+	--	--	-	+
**DOX**	-	-	-	+/-	-	+	-	+
**LZD**	-	-	-	-	-	-	+	+
**CIP**	-	-	+	-	-	-	-	+
**IMP**	--	-	-	-	-	-	-	+
**VAN**	-	-	--	-	--	-	-	+
**AMP**	-	-	-	-	-	-	-	+/-
**DAP**		-	--	+/-		-	-	+/-
**Q-D**	-		ND	ND	-		ND	ND

^1^ Disk diffusion; GEN, gentamycin; STR, streptomycin; TGC, tigecycline; DOX, doxycycline; LZD, linezolid; CIP, ciprofloxacin; IMP, imipenem; VAN, vancomycin; AMP, ampicillin; DAP, daptomycin; Q-D, quinupristin-dalfopristin (Synercid); PAE, post antibiotic effect; FL, fullerene; RB, rose bengal; ND, not defined. (+), synergy; (+/-), partial synergy; (-) no synergistic effect; (--) antagonism.

**Table 5:** Summarized results of synergy testing for *E. faecalis*.

Antibiotic	aPDI (RB)				aPDI (FL)			
	DF ^1^	E-Test	Checkerboard Assay	PAE	DF	E-Test	Checkerboard Assay	PAE
**GEN**	-	-	+	-	-	-	-	+
**STR**	+	+	-	+	+	+	-	+
**TGC**	+	+	-	+	+	--	-	+
**DOX**	+/-	+	-	+	-	+	-	+
**LZD**	-	-	-	-	-	-	-	+
**CIP**	-	-	+	-	+	-	-	+
**IMP**	+/-	-	-	-	-	-	+	+
**VAN**	-	-	-	-	-	--	+	+
**AMP**	+	-	-	+	+	-	-	-
**DAP**		-	+	-			-	+
**Q-D**	-		ND	ND	-	-	ND	ND

^1^ Disk diffusion; GEN, gentamycin; STR, streptomycin; TGC, tigecycline; DOX, doxycycline; LZD, linezolid; CIP, ciprofloxacin; IMP, imipenem; VAN, vancomycin; AMP, ampicillin; DAP, daptomycin; Q-D, quinupristin-dalfopristin (Synercid); PAE, post antibiotic effect; FL, fullerene; RB, rose Bengal; ND, not defined; (+), synergy; (+/-), partial synergy; (-) no synergistic effect; (--) antagonism.

## Data Availability

The data presented in this study are available on request from the corresponding author.

## References

[1] Růžičková M., Vítězová M., Kushkevych I. (2020). The characterization of Enterococcus genus: Resistance mechanisms and inflammatory bowel disease. Open Med..

[2] Massier S., Bouffartigues E., Rincé A., Maillot O., Feuilloley M.G.J., Orange N., Chevalier S. (2013). Effects of a pulsed light-induced stress on Enterococcus faecalis. J. Appl. Microbiol..

[3] Nakonieczna J., Wozniak A., Pieranski M., Rapacka-Zdonczyk A., Ogonowska P., Grinholc M. (2019). Photoinactivation of ESKAPE pathogens: Overview of novel therapeutic strategy. Future Med. Chem..

[4] Cheng X., Tian Y., Zhao C., Qu T., Ma C., Liu X., Yu Q. (2016). Bactericidal effect of strong acid electrolyzed water against flow enterococcus faecalis biofilms. J. Endod..

[5] Cieplik F., Deng D., Crielaard W., Buchalla W., Hellwig E., Al-Ahmad A., Maisch T. (2018). Antimicrobial photodynamic therapy—What we know and what we don’t. Crit. Rev. Microbiol..

[6] Maisch T., Hackbarth S., Regensburger J., Felgenträger A., Bäumler W., Landthaler M., Röder B. (2011). Photodynamische inaktivierung von multiresistenten bakterien (PIB)—Ein neuer ansatz zur behandlung oberflächlicher infektionen im 21. jahrhundert. JDDG J. Ger. Soc. Dermatology.

[7] Pidot S.J., Gao W., Buultjens A.H., Monk I.R., Guerillot R., Carter G.P., Lee J.Y.H., Lam M.M.C., Grayson M.L., Ballard S.A. (2018). Increasing tolerance of hospital *Enterococcus faecium* to handwash alcohols. Sci. Transl. Med..

[8] Sassoubre L.M., Nelson K.L., Boehm A.B. (2012). Mechanisms for photoinactivation of Enterococcus faecalis in seawater. Appl. Environ. Microbiol..

[9] Vatansever F., de Melo W.C.M.A., Avci P., Vecchio D., Sadasivam M., Gupta A., Chandran R., Karimi M., Parizotto N.A., Yin R. (2013). Antimicrobial strategies centered around reactive oxygen species—Bactericidal antibiotics, photodynamic therapy, and beyond. FEMS Microbiol. Rev..

[10] Hamblin M.R., Viveiros J., Yang C., Ahmadi A., Ganz R.A., Tolkoff M.J. (2005). Helicobacter pylori accumulates photoactive porphyrins and is killed by visible light. Antimicrob. Agents Chemother..

[11] Wozniak A., Rapacka-Zdonczyk A., Mutters N.T., Grinholc M. (2019). Antimicrobials Are a Photodynamic Inactivation Adjuvant for the Eradication of Extensively Drug-Resistant Acinetobacter baumannii. Front. Microbiol..

[12] Magiorakos A.-P., Srinivasan A., Carey R.B., Carmeli Y., Falagas M.E., Giske C.G., Harbarth S., Hindler J.F., Kahlmeter G., Olsson-Liljequist B. (2012). Multidrug-resistant, extensively drug-resistant and pandrug-resistant bacteria: An international expert proposal for interim standard definitions for acquired resistance. Clin. Microbiol. Infect..

[13] Liu Y., Imlay J.A. (2013). Cell death from antibiotics without the involvement of reactive oxygen species. Science.

[14] Grinholc M., Nakonieczna J., Fila G., Taraszkiewicz A., Kawiak A., Szewczyk G., Sarna T., Lilge L., Bielawski K.P. (2015). Antimicrobial photodynamic therapy with fulleropyrrolidine: Photoinactivation mechanism of Staphylococcus aureus, in vitro and in vivo studies. Appl. Microbiol. Biotechnol..

[15] Paganelli F.L., Willems R.J.L.W., Jansen P., Hendrickx A., Zhang X., Bonten M.J.M.B., Leavis H.L. (2013). Enterococcus faecium biofilm formation: Identification of major autolysin AtlAefm, associated acm surface localization, and AtlAefm-independent extracellular DNA release. MBio.

[16] Zhang C., Du J., Peng Z. (2015). Correlation between Enterococcus faecalis and Persistent Intraradicular Infection Compared with Primary Intraradicular Infection: A Systematic Review. J. Endod..

[17] Tavares A., Carvalho C., Faustino M.A., Neves M.G., Tomé J.P., Tomé A.C., Cavaleiro J.A., Cunha Â., Gomes N., Alves E. (2010). Antimicrobial photodynamic therapy: Study of bacterial recovery viability and potential development of resistance after treatment. Mar. Drugs.

[18] Radunović M., Petrini M., Vlajic T., Iezzi G., Di Lodovico S., Piattelli A., D’Ercole S. (2020). Effects of a novel gel containing 5-aminolevulinic acid and red LED against bacteria involved in peri-implantitis and other oral infections. J. Photochem. Photobiol. B Biol..

[19] Chibebe J., Fuchs B.B., Sabino C.P., Junqueira J.C., Jorge A.O.C., Ribeiro M.S., Gilmore M.S., Rice L.B., Tegos G.P., Hamblin M.R. (2013). Photodynamic and Antibiotic Therapy Impair the Pathogenesis of Enterococcus faecium in a Whole Animal Insect Model. PLoS ONE.

[20] Kang S.M., Jung H.I., Kim B.I. (2019). Susceptibility of oral bacteria to antibacterial photodynamic therapy. J. Oral Microbiol..

[21] López-Jiménez L., Fusté E., Martínez-Garriga B., Arnabat-Domínguez J., Vinuesa T., Viñas M. (2015). Effects of photodynamic therapy on Enterococcus faecalis biofilms. Lasers Med. Sci..

[22] Garcez A.S., Núñez S.C., Azambuja N., Fregnani E.R., Rodriguez H.M.H., Hamblin M.R., Suzuki H., Ribeiro M.S. (2013). Effects of photodynamic therapy on gram-positive and gram-negative bacterial biofilms by bioluminescence imaging and scanning electron microscopic analysis. Photomed. Laser Surg..

[23] Fonseca M.B., Tessare P.O., Pallota R.C., Filho H.F., Denardin O.V.P., Rapoport A., Dedivitis R.A., Veronezi J.F., Genovese W.J., Ricardo A.L.F. (2008). Photodynamic therapy for root canals infected with Enterococcus faecalis. Photomed. Laser Surg..

[24] Shrestha A., Hamblin M.R., Kishen A. (2012). Characterization of a conjugate between rose bengal and chitosan for targeted antibiofilm and tissue stabilization effects as a potential treatment of infected dentin. Antimicrob. Agents Chemother..

[25] Manoil D., Filieri A., Schrenzel J., Bouillaguet S. (2016). Rose bengal uptake by E. faecalis and F. nucleatum and light-mediated antibacterial activity measured by flow cytometry. J. Photochem. Photobiol. B Biol..

[26] Wozniak A., Grinholc M. (2018). Combined antimicrobial activity of photodynamic inactivation and antimicrobials-state of the art. Front. Microbiol..

[27] Miller W.R., Munita J.M., Arias C.A. (2014). Mechanisms of antibiotic resistance in enterococc. Expert Review of Anti-Infective Therapy.

[28] Fiedler S., Bender J.K., Klare I., Halbedel S., Grohmann E., Szewzyk U., Werner G. (2016). Tigecycline resistance in clinical isolates of Enterococcus faecium is mediated by an upregulation of plasmid-encoded tetracycline determinants tet(L) and tet(M). J. Antimicrob. Chemother..

[29] Gagetti P., Bonofiglio L., García Gabarrot G., Kaufman S., Mollerach M., Vigliarolo L., von Specht M., Toresani I., Lopardo H.A. (2019). Resistance to β-lactams in enterococci. Rev. Argent. Microbiol..

[30] Ono S., Muratani T., Matsumoto T. (2005). Mechanisms of resistance to imipenem and ampicillin in Enterococcus faecalis. Antimicrob. Agents Chemother..

[31] Dwyer D.J., Belenky P.A., Yang J.H., MacDonald I.C., Martell J.D., Takahashi N., Chan C.T., Lobritz M.A., Braff D., Schwarz E.G. (2014). Antibiotics induce redox-related physiological alterations as part of their lethality. Proc. Natl. Acad. Sci. USA.

[32] Bandyopadhyay D., Mukherjee M. (2020). Reactive oxygen species and uspA overexpession: An alternative bacterial response toward selection and maintenance of multidrug resistance in clinical isolates of uropathogenic E. coli. Eur. J. Clin. Microbiol. Infect. Dis..

[33] Memar M.Y., Ghotaslou R., Samiei M., Adibkia K. (2018). Antimicrobial use of reactive oxygen therapy: Current insights. Infect. Drug Resist..

[34] Pieranski M., Sitkiewicz I., Grinholc M. (2020). Increased photoinactivation stress tolerance of Streptococcus agalactiae upon consecutive sublethal phototreatments. Free Radic. Biol. Med..

[35] Rapacka-Zdonczyk A., Wozniak A., Pieranski M., Woziwodzka A., Bielawski K.P., Grinholc M. (2019). Development of Staphylococcus aureus tolerance to antimicrobial photodynamic inactivation and antimicrobial blue light upon sub-lethal treatment. Sci. Rep..

[36] Di Poto A., Sbarra M.S., Provenza G., Visai L., Speziale P. (2009). The effect of photodynamic treatment combined with antibiotic action or host defence mechanisms on Staphylococcus aureus biofilms. Biomaterials.

